# Modelling the case for cost-effectiveness of interventions to improve medication adherence in patients with difficult to control asthma

**DOI:** 10.3310/nihropenres.13775.1

**Published:** 2025-01-27

**Authors:** Dacheng Huo, Sebastian Hinde, Harriet Smith, David Linden, Llinos Jones

**Affiliations:** 1 University of York Centre for Health Economics, York, England, UK; 2 Health Innovation Yorkshire & Humber, Wakefield, UK; 3 Mid Yorkshire Teaching NHS Trust, Wakefield, UK

**Keywords:** Asthma Guidelines, Asthma in primary care, Health Economist

## Abstract

**Background:**

Asthma is a prevalent non-communicable disease in high-income countries, affecting more than 10% of their populations. While often symptomatically mild it accounts for 2-3% of primary care appointments, 60,000 hospital admissions, and 1,200 deaths annually in England. Importantly, a significant proportion of these events are the result of poor adherence to prescribed and effective treatments, with 60% of hospital admissions attributed to suboptimal compliance. This study aims to support the development and commissioning of interventions by establishing the value case for improving medication adherence.

**Methods:**

A probabilistic cohort decision model was developed with the aim of estimating the long-term health impacts and resultant costs to the healthcare system of different levels of population medication adherence for those with difficult-to-control asthma. The model applies a Markov structure based on the Global Initiative for Asthma (GINA) guidelines to define the level of asthma control. Informative parameters are drawn from existing published literature supplemented by expert input where required.

**Results:**

Improved adherence is associated with reduced asthma exacerbations and better overall health of the cohort, measured in life years and quality-adjusted life years (QALYs). For instance, an educational intervention that increased adherence from 50% to 70% reduced exacerbations by 1.75 over 20 years, while increasing QALYs by 0.20, and reducing the healthcare costs by £989.

**Conclusions:**

Significant economic and health benefits can be achieved with effective interventions to improve treatment adherence in asthma. This study provides a value case for developing and commissioning such interventions.

## Plain language summary

**Key messages:**

**What is already known on this topic**

•    Despite the existence of extensive treatment options for asthma available many people still require hospitalisation and die as a result of the disease, often as a result of poor adherence to prescribed medication.

•    The effectiveness and cost-effectiveness of many individual treatments for asthma is well known but these are typically based on optimal adherence being achieved, with limited research available on the impact of poor adherence or the value case for investing in interventions to address it.

**What this study adds - summarise what we now know as a result of this study that we did not know before**

•    This study provides quantitative evidence of the health and care implications of different levels of asthma medication adherence, demonstrating the potential health gains and healthcare saving that can be achieved.

**How this study might affect research, practice or policy**

•    Working collaboratively between academic, clinical and policy experts this study provides a methodologically and clinically robust accessible guide for key commissioning stakeholders to support steps towards improving medication adherence in asthma in a cost-effective way.

## Background and objectives

Asthma represents a significant public health challenge in the United Kingdom, affecting approximately 12% of the population, or roughly 8 million individuals each year. The country witnesses 160,000 new cases each year, underscoring the pervasive nature of this chronic respiratory condition ( NICE, 2021). Asthma also accounts for approximately 1,200 deaths annually in England, as reported by the Office for National Statistics ( Statistics, 2021a). It has a substantial impact on the healthcare system, accounting for 2–3% of primary care appointments, 60,000 hospital admissions and 200,000 bed days per year ( NICE, 2021) and the resulting inhaler medication is a key contributor to the National Health Service (NHS) carbon footprint ( NHS England, 2022). The absence of a cure for asthma accentuates the critical role of effective medication prescribing and, crucially, patient adherence to inhaler regimens. These measures are vital for symptom management and exacerbation prevention.

Despite the clear benefits of adherence, achieving consistent patient compliance with asthma therapies poses a significant hurdle. A detailed qualitative systematic review has illuminated various barriers that patients encounter, hindering their treatment adherence ( Dima *et al.*, 2015). Notably, research by Barnes and Ulrik links poor adherence to an increased frequency of acute asthma episodes, with 24% of flare-ups and 60% of hospital admissions attributed to suboptimal treatment compliance ( Bårnes & Ulrik, 2015). These incidents are notably influenced by socio-economic factors ( Mazumdar *et al.*, 2015), suggesting a complex interplay of systemic issues affecting patient adherence.

In light of these challenges, our study aims to assess the long-term health impact and resultant cost to the health and social care system of poor adherence to treatment, and by extension a means of estimating the potential cost-effectiveness of interventions designed to enhance adherence. To do so we developed a cohort decision model to evaluate the long-term repercussions of sub-optimal adherence on patient health outcomes and the financial burden on the NHS. Recognizing the dynamic nature of healthcare environments and the intricacies of intervention contexts, our model is designed for flexibility. It accommodates different baseline adherence levels, enabling policymakers to derive tailored, practical insights for decision-making. Our objective is to deliver a robust analytical framework that not only captures the economic dimensions of adherence interventions but also addresses the needs of policymakers in a real-world setting.

## Methods

### Patient and Public Involvement (PPI)

While the analysis reported in this paper is based on a hypothetical cohort of patients PPI formed a fundamental part of the inspiration of the research idea. Data collected from interviews and focus groups, conducted as part of the Straight Talking Asthma Information Project, suggested that there was a variable level of knowledge about asthma care, with many myths and misconceptions circulating around the condition.

Interviews from the South Asian community in Kirklees uncovered that language played an enormous role in community members not knowing where to access help for asthma, including what to do in an emergency situation. In some cases there were low levels of understanding when attending doctors’ appointments, how and when to take medications. Community members also stated that they often felt excluded and under- represented in the health literature that is available to them. Much health literature did not visibly represent them, nor did they cover the culturally specific questions that they wished to ask.

These insights from patients and communities directly led to the collaboration between the authors of this paper and the underlying aim of this study to better understand the value case for investment in interventions which improve the adherence to asthma medication.

### The model overview

**Figure 1.**
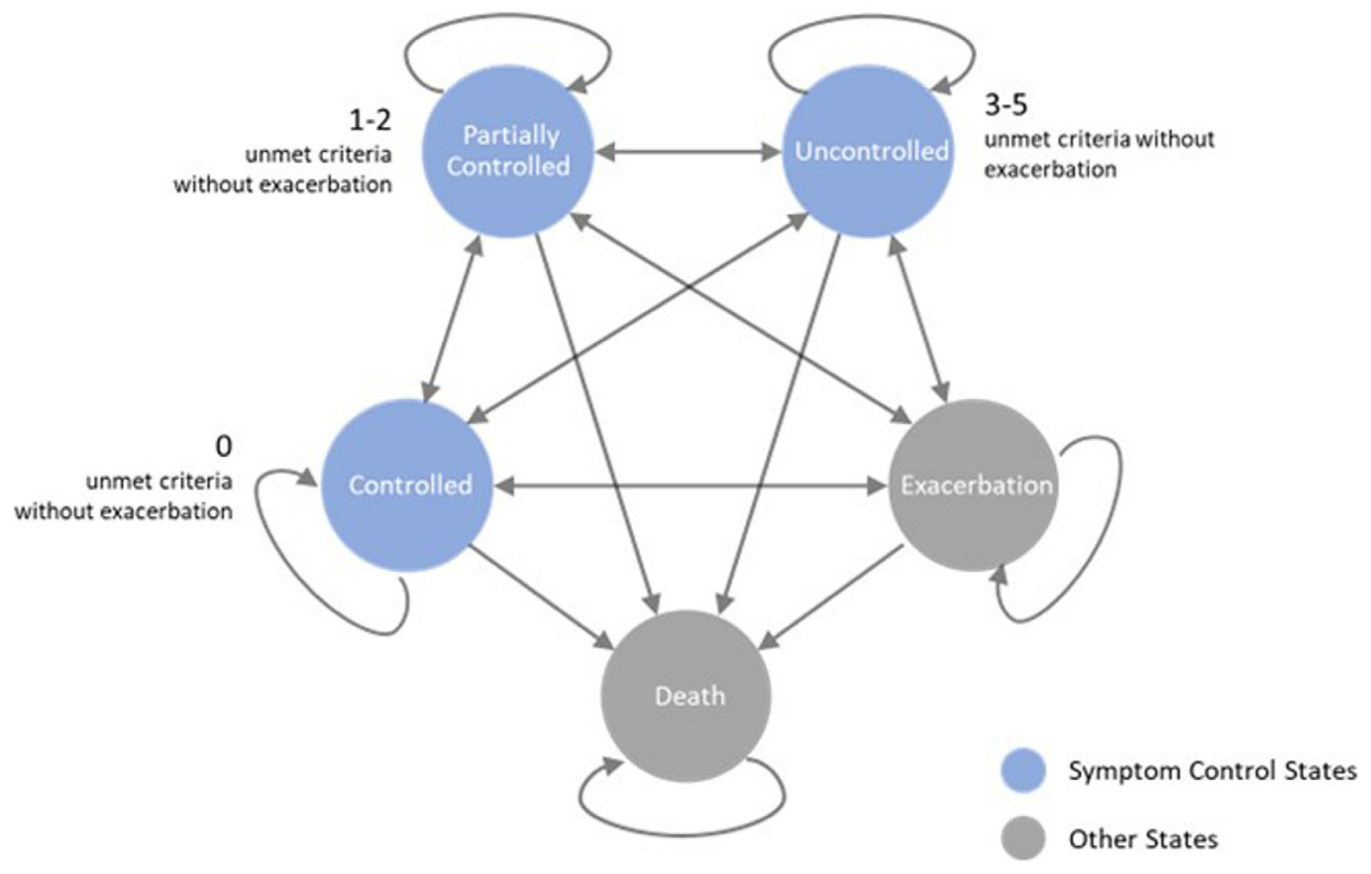
Schematic representation of the modified Asthma Markov Model.

This research employs a refined Markov model from Zafari’s study ( Zafari *et al.*, 2016), which categorizes patients into three symptom control states—controlled, partially controlled, and uncontrolled—alongside states for exacerbation and death. We extend its application to examine asthma management dynamics over a 20-year horizon, using weekly cycles to capture short-term changes and long-term outcomes. Asthma control criteria are aligned with the Global Initiative for Asthma (GINA) guidelines ( Asthma, 2023). Notably, the model accounts for asthma-related mortality exclusively in the context of exacerbations, distinguishing other deaths as non-asthma related. Figure 1 illustrates the operational structure of the model, delineating the transitions between different states of asthma control, exacerbation, and mortality. The model's computations are executed within Excel.

Asthma control, as per GINA guidelines, is assessed weekly through a combination of patient diary entries and exacerbation data, focusing on: (1) absence of night-time symptoms, (2) two or fewer days of daytime symptoms per week, (3) limited reliever usage, (4) maintaining Peak Expiratory Flow at or above 80% of the predicted normal, and (5) no limitations on daily activities. A week is classified as 'Controlled' if all criteria are met without severe exacerbation. 'Partially Controlled' weeks feature one or two unmet criteria without exacerbation, while weeks with three or more unmet criteria or any exacerbation are deemed 'Uncontrolled.'

### Cohort patients

The study specifically addresses adults with difficult-to-control asthma from the NHS and personal social services (PSS) perspective, recognizing the heightened risk and crucial need for adherence in this subgroup ( Murphy *et al.*, 2012). Utilizing data from the 2020 Annual Asthma Survey ( Asthma UK, 2020), the initial state distribution reflects the current UK asthma population: 40.1% uncontrolled, 39.4% partly controlled, and 20.5% fully controlled. This baseline facilitates an accurate assessment of intervention impacts under prevailing conditions. The study further categorizes exacerbations by severity and hospital admission status—39% admitted versus 61% non-admitted—highlighting the diverse management needs within the difficult-to-control asthma demographic ( Losappio *et al.*, 2019).

### Model assumptions

Three key assumptions underlie our model's design:

1) Exacerbation Duration and Hospital Stays: The length of acute exacerbations and associated hospitalizations does not affect transition probabilities outside of control status and medication adherence. This assumption simplifies the model without compromising its ability to capture critical dynamics.

2) Constant Adherence Levels: Adherence is considered static over time for each patient, despite potential real-world variations influenced by symptom severity and exacerbation frequency. This facilitates a straightforward comparison of long-term outcomes across different adherence scenarios.

3) Exclusivity of Exacerbation Severity: While patients may experience multiple exacerbation types within a single cycle, the model treats exacerbation severity as mutually exclusive. This approach allows for a direct comparison between cohorts with varying adherence levels, elucidating the distinct long-term outcomes tied to adherence.

### Transition probabilities and treatment effectiveness

The transition probabilities within the Markov model were derived from a meta-analysis conducted by Chongmelaxme ( Chongmelaxme *et al.*, 2020), focusing on the odds ratio of care discontinuity. The non-asthma related mortality rates were sourced from the National Life Tables by the Office for National Statistics of the United Kingdom ( Statistics, 2021b). Given that asthma severity fluctuates but does not directly correlate with mortality in the context of this study, no differential mortality was assumed between the health states other than via correlation with exacerbations.

Under scenarios of full adherence, the transition probabilities among the three asthma control states and exacerbation rates were informed by Bateman ( Bateman *et al.*, 2010), while mortality rates specific to asthma exacerbations were adopted ( Engelkes *et al.*, 2020). Transitions from the exacerbation state back to the control states were based on findings from Bateman ( Bateman *et al.*, 2008). Under scenarios of zero adherence (i.e. no treatment), the probabilities of transition were calculated using odds ratios from Chongmelaxme's meta-analysis ( Chongmelaxme *et al.*, 2020), providing a comprehensive understanding of care discontinuity impacts on asthma management.

**Figure 2.**
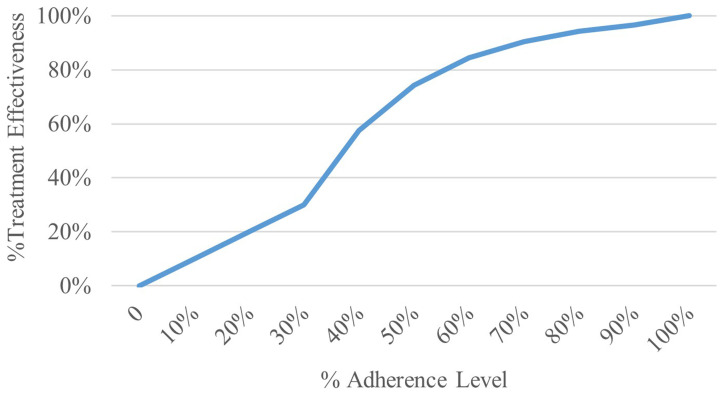
Treatment effectiveness by adherence level.

This study assumes a non-linear relationship between treatment adherence and effectiveness, where effectiveness is exponentially related to adherence rates. This allows for a nuanced comparison of intervention cost-effectiveness across varying adherence levels, modelled after the systematic review and meta-analysis by ( Chongmelaxme & Dilokthornsakul, 2019). Figure 2 illustrates the relationship between treatment adherence and effectiveness, serving as a visual aid to complement our analytical model. The formula used to calculate treatment effectiveness is as follows:

- For adherence rates ≤ 30%, treatment effectiveness is directly proportional to the adherence rate.
- For adherence rates > 30% and < 100%, effectiveness is calculated using the formula: 1 – exp ( -5 * (%adherence rate – 0.2287))
- An adherence rate of 100% correlates with a treatment effectiveness of 100%.

### Health resource utilization and model costs

Healthcare resource utilization and associated costs were catalogued, encompassing prescription refills for maintenance therapy, outpatient visits, and rescue therapy for patients across all asthma control states. Exacerbation care was differentiated between non-admitted and admitted patients, with healthcare utilization tailored to each scenario, including outpatient attendance, community asthma care services, and medication costs for non-admitted patients, and outpatient service, hospitalization, and ambulance services for admitted patients. These costs were aligned with recent national unit cost data, ensuring an accurate economic analysis. All costs are presented in 2022 GBP (Great British Pounds).

The model acknowledges variations in patient treatment steps, as defined by the National Institute for Health and Care Excellence ( NICE, 2021), to tailor treatment intensity and minimize unnecessary medication use while maintaining asthma control. This approach is based on an observational study by ( Bloom *et al.*, 2020), which provided insights into treatment step distribution within the population.

Outpatient attendance rates were estimated based on GINA 2023 recommendations ( Asthma, 2023), with well-controlled patients assumed to visit every six months, partially controlled patients every three months, and uncontrolled patients monthly. The unit costs for medication refills were sourced from the NICE website and NHS Reference Costs 2021/22 ( NHS, 2023). This study's scope specifically addresses adherence and its effectiveness in relation to pharmacological management, excluding the variable of outpatient visit frequency as influenced by medication adherence levels from the analysis. Consequently, the model differentiates the impact of adherence levels solely on maintenance therapy-related healthcare resource utilization. In contrast, resource use for rescue therapy and exacerbation treatment remains consistent across adherence levels, reflecting the expectation that patients with poor adherence will still seek emergency care in severe conditions.

### Health state utility values and cost-effectiveness analysis

Health state utility values were adopted ( Briggs *et al.*, 2021), with HRQoL measured using the EQ-5D-3L instrument, adjusted for age and sex based on the Health Survey for England ( Craig *et al.*, 2013). Outcomes were quantified in quality-adjusted life years (QALYs), incorporating utility data for both non-admitted (61%) and admitted (39%) patients.

Results are presented related to the impact of changes to the adherence rate on the mean values of the key outputs of the decision analytic model, specifically number of exacerbations, life years, QALYs, and total costs to the NHS and PSS. Consistent with best practice for conducting comparative cost-effectiveness analysis in the UK, defined by the NICE methods guide, all outputs are reported over a 20-year time horizon but with costs and QALYs are discounted at a rate of 3.5% per annum ( NICE, 2022).

To assess the robustness of our model's outcomes against uncertainties in parameter estimates, a probabilistic sensitivity analysis (PSA) was conducted. This analysis adheres to the methodological framework proposed by Briggs *et al.* ( Briggs *et al.*, 2006), which recommends a comprehensive approach to examining the impact of parameter uncertainty on model results. Specifically, the model was configured to execute 1,000 Monte Carlo simulations for each of the 10 levels of medication adherence reported, encapsulating the variability in key parameters. Distributional assumptions for these parameters were selected based on best practices outlined in the cited guidelines, ensuring that the analysis reflects realistic variations in model inputs, a full list of the distributions used, and the informative sources and assumptions, is available in the Appendix. For each of the four key model outputs the mean cohort values are reported alongside a 95% confidence interval drawn from the Monte-Carlo simulation.

## Results

**Figure 3.**
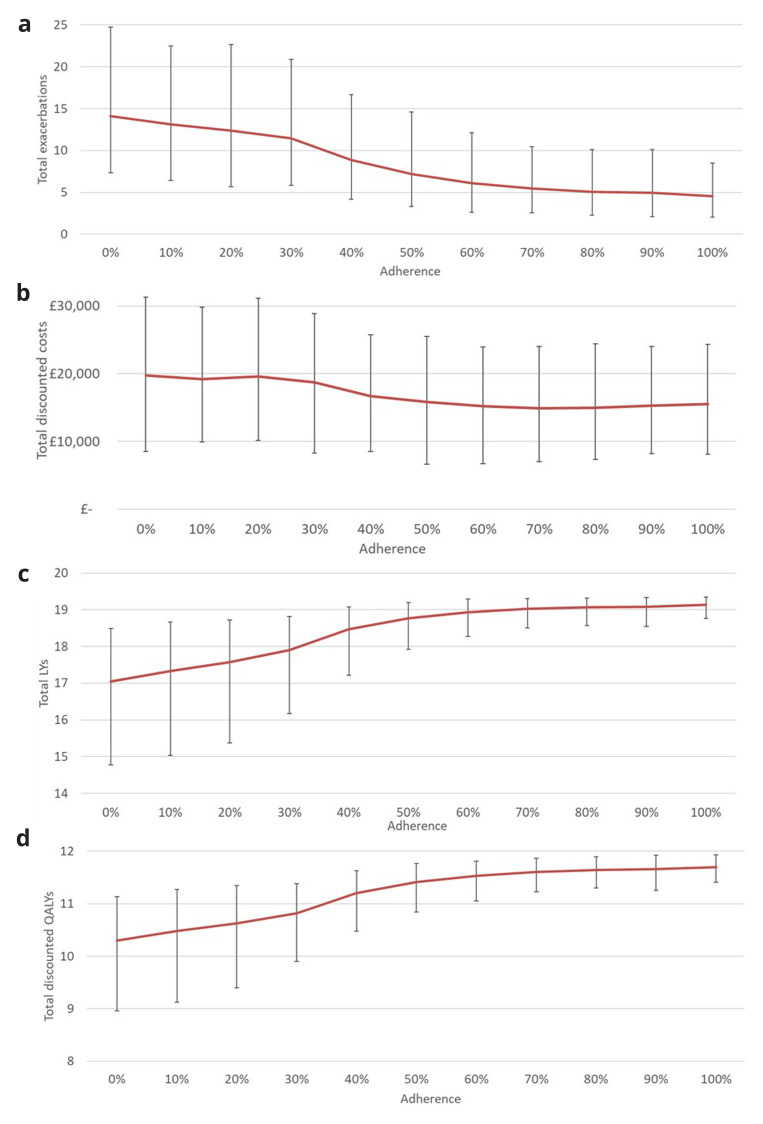
( **a**) total exacerbations over 20 years by adherence percentage, ( **b**) total discounted costs over 20 years by adherence percentage, ( **c**) total life-years over 20 years by adherence percentage, ( **d**) total discounted QALYs over 20 years by adherence percentage.

The mean cohort results from the probabilistic decision model across the four key outputs, number of exacerbations, discounted costs, life years, and discounted QALYs, are reported below in Figure 3a, 3b, 3c, and 3d, alongside 95% confidence intervals.

As expected, a-priori, increasing levels of medical adherence decreases the estimated number of exacerbations over the 20-year time-horizon, this and other factors results in a corresponding increase in life-years and QALYs. The non-linear trend in these outputs is the result of the similar trend modelled between medical adherence level and their effectiveness described in Figure 2. In contrast, the relationship between adherence level and discounted costs is more complex, with changes in adherence having limited difference on costs at both extremes, i.e. between 0%–30% and 60%–100%. In between these regions there is, however, a negative relationship between adherence and costs. The reason for this is there are two opposing factors influencing total costs, the cost of the medication which increases as adherence does, and the cost of treatment asthma related events including exacerbations which decrease as adherence increases. The opposing elements are relatively balanced at the extremes of adherence but between 30% and 60% the increased impact of changes in adherence with treatment effectiveness dominate the increased cost of the medication.

The Figures 3a–d also provide the ability to directly compare the estimated impact of increasing levels of adherence, for example as the result of a new intervention. For instance, an educational intervention that increased an individual’s medication adherence from 50% to 70% would be expected to reduce their number of exacerbations by 1.75 over 20 years, while increasing their expected life years by 0.26 years, discounted QALYs by 0.20, while reducing the discounted costs of providing their healthcare by £989.

The figures also show that there are high levels of uncertainty across all of the outputs. The model indicated that this uncertainty reduces as adherence increases as relatively less is known about the scale of impact of reduced adherence levels on asthma events and therefore the long-term health and associated costs.

## Discussion

The findings from our analyses highlight the substantial benefits that can be realized through improved medication adherence among asthma patients. By enhancing adherence, not only can patient health outcomes be significantly improved, but the economic burden on healthcare systems can also be alleviated. The predicted reduction in exacerbations should also reduce the number of GP appointments, emergency department presentations and hospital admissions, and therefore help to alleviate workforce pressures across the system.

Improving adherence to preventer treatments can have further positive impacts, including reducing the number of reliever inhalers used and thus supporting the NHS ambition to become net zero. By reducing the number of reliever inhalers, there is a corresponding reduction in the carbon footprint associated with manufacturing, transporting and disposing of these devices, plus a reduction in emissions from the propellant in metered-dose inhalers (MDIs) which is often used to deliver the medicine.

These results provide a compelling argument for the development and implementation of targeted, personalised interventions aimed at increasing medication adherence rates, with the potential to yield meaningful improvements in patient well-being, healthcare resource utilization, and environmental sustainability.

This study acknowledges several limitations that may impact the generalizability and applicability of its findings. Firstly, the model's capacity to generalize adherence outcomes across different populations is constrained by its design, which does not fully account for the myriad scenarios and contextual factors influencing adherence improvement interventions. Such factors include varying healthcare systems, patient demographics, levels of health literacy, and the multifaceted nature of patient engagement with treatment regimens. Furthermore, the study recognizes the challenge of diverse definitions and measures of adherence levels across the existing body of research. While to some extent these elements of uncertainty are incorporated in the analysis through the PSA, such an analysis is only able to incorporate uncertainty that can be accurately quantified. For example, we found little evidence regarding the correlation between control status and required frequency of outpatient appointments, which due to the high cost of such appointments was found to be a significant driver of the estimated cost of providing healthcare to this population.

Despite these limitations, the study presents a novel and flexible model for estimating the cost-effectiveness of various interventions aimed at improving medication adherence. This model is particularly relevant within the context of patient- or user-centred research, emphasizing the importance of designing healthcare interventions that are tailored to the needs and preferences of patients. As such, it serves as a valuable resource for policymakers, healthcare providers, and carers, facilitating informed decision-making regarding the allocation of resources toward adherence-enhancing strategies.

Previous studies have identified the expected cost-effectiveness of medication adherence enhancing interventions for asthma ( Khaw *et al.*, 2022) including two relatively old studies in a UK setting ( Drummond *et al.*, 1994; Pinnock *et al.*, 2007). However, these only consisted of studies of individual studies of varying quality. In contrast this study demonstrates the potential value of optimising adherence and facilitates a consistent approach to the valuation of such interventions from a cost-effectiveness viewpoint.

## Conclusion

The model's findings highlight the potential economic and health benefits of increasing treatment adherence in asthma management, reinforcing the argument for interventions that are not only clinically effective but also cost-efficient. Such interventions could include community-based programs, policy reforms aimed at reducing healthcare costs for underprivileged populations, and educational campaigns designed to increase asthma self-management practices and health literacy. However, the significant uncertainty in all of the outputs from the analysis demonstrates that further research is needed to provide a better understanding of not only how routine medication impacts asthma related health over the long-term, but importantly how less than optimal adherence to prescribed medication correlates with these impacts.

## Ethics approval and data sharing

Not applicable, this study does not involve human participants, animal subjects, or clinical trials. It does not contain any personal or medical information about identifiable individuals, nor does it include any case reports or case histories of identifiable individuals.

## Third party ethics

All data used in our study were derived from publicly available, open-access sources and did not require additional ethical approval or consent. Public datasets comply with ethical standards at the point of collection and are made available for research without individual consent, aligning with data-sharing protocols.

## AI use statement

Not applicable, not applicable, no AI is used in this study

## Data Availability

**Third party data** Figshare: Modelling the case for cost-effectiveness of interventions to improve medication adherence in patients with difficult to control asthma ( Huo *et al.*, 2024) DOI: https://doi.org/10.6084/m9.figshare.27828765 The project contains the following underlying data: Supplementary Materials - Full list of model parameters which should includes the sources and third-party data used in the study. Assumptions made are also noted where applicable. Data are available under the terms of the Creative Commons Attribution 4.0 International license (CC-BY 4.0). Figshare: Modelling the case for cost-effectiveness of interventions to improve medication adherence in patients with difficult to control asthma ( Huo *et al.*, 2024) DOI: https://doi.org/10.6084/m9.figshare.27828765 The project contains the following underlying data: Figure 1: Schematic representation of the modified Asthma Markov Model Figure 2: Treatment effectiveness by adherence level Figure 3a: total exacerbations over 20 years by adherence percentage Figure 3b: total discounted costs over 20 years by adherence percentage Figure 3c: total life-years over 20 years by adherence percentage Figure 3d: total discounted QALYs over 20 years by adherence percentage Data are available under the terms of the Creative Commons Attribution 4.0 International license (CC-BY 4.0).
