# Supplementary material for: Modelling the case for cost-effectiveness of interventions to improve medication adherence in patients with difficult to control asthma
Source: NIHR Open Res. 2025 Oct 17;5:8. Originally published 2025 Jan 27. [Version 2] doi: 10.3310/nihropenres.13775.2 (PMC12627936; doi:10.3310/nihropenres.13775.2)
Supplement: Peer review history

# Reviewer report: Reviewer response for version 2

**DOI:** 10.3310/nihropenres.15379.r38109

**Published:** 2025-11-22

**Authors:** Giovanna Culeddu

**Affiliations:** 1 Bangor University, Bangor, UK

Thank you for the opportunity to review this manuscript. While the paper addresses an important and timely topic — the impact of medication adherence on asthma outcomes and health system costs — I regret to conclude that the manuscript in its current form does not meet the methodological or reporting standards required for a robust cost-effectiveness analysis.

 The title and stated objectives suggest a formal economic evaluation of interventions to improve adherence. However, the work instead presents a hypothetical scenario analysis of adherence levels, without defining, modelling, or comparing actual interventions. This disconnect between the title, objective, and analytical content is problematic and undermines the study’s credibility and utility.

 Major Concerns

 1.    Misrepresentation of Study Type

 The title and abstract strongly imply a cost-effectiveness analysis of real-world interventions (e.g., education programmes, digital tools, policy reforms). However, the study does not specify or compare any such interventions. Instead, it models theoretical shifts in adherence percentages and projects the downstream health and cost consequences. This is more appropriately described as a scenario modelling study, not a cost-effectiveness analysis.

 2.    Absence of Key Health Economic Outputs

 Despite claiming a cost-effectiveness modelling framework, the paper does not report:

 o    Incremental Cost-Effectiveness Ratios (ICERs)

 o    Incremental Net Benefits (INBs)

 o    Cost-effectiveness acceptability curves (CEACs)

 o    Threshold-based decision metrics (e.g. comparisons to £20,000–£30,000 per QALY)

 These are all expected in evaluations intended to inform resource allocation decisions, especially under NICE guidance.

 3.    No Definition of Interventions or Comparators

 The manuscript lacks any description of the specific treatments, adherence-improvement interventions, or implementation strategies being evaluated. As a result, it is not possible to understand or replicate what is being compared. The claim that the model can inform decision-makers is undermined by this lack of clarity.

 4.    Incomplete Cost and Utility Reporting

 There is no transparent breakdown of cost or utility inputs by health state. The paper mentions using EQ-5D data and NHS Reference Costs but fails to present the specific utility values or healthcare resource use assumptions per state or event (e.g. exacerbations, outpatient visits, medication use). This compromises both transparency and credibility.

 5.    Flawed Model Assumptions

 A particularly concerning assumption is that 100% adherence leads to 100% treatment effectiveness, and by extension, the complete absence of asthma exacerbations. This is clinically implausible. Real-world evidence consistently shows that even fully adherent patients — particularly those with severe or eosinophilic asthma — continue to experience exacerbations. This assumption likely leads to overestimation of benefits and misrepresents the true burden of disease.

 6.    Model Structure Does Not Reflect Stated Mortality Assumptions

 While the manuscript states that asthma-related mortality is exclusively modelled via exacerbations, the schematic Markov model contradicts this, as it allows direct transitions to death from all asthma control states. This inconsistency raises concerns about the internal coherence of the model structure and the assumptions underpinning the mortality estimates.

 7.    Assumption of Static Adherence

 The model assumes constant adherence over time, which is not reflective of real-world dynamics, where adherence often decays or fluctuates. While this is acknowledged as a limitation, it has not been addressed methodologically — despite the availability of adherence decay models in the literature.

 8.    Over-simplification of Exacerbation Risk

 The model treats exacerbation severity as mutually exclusive within a given cycle. However, patients often experience multiple and varied exacerbations in close succession, and this simplification may underestimate cumulative burden and costs.

 9.    Population Homogeneity

 The model does not stratify results by age, sex, asthma phenotype, or comorbidities. These omissions reduce the model's applicability to clinical subgroups and diminish its policy relevance.

 10.    Absence of Structural and Parameter Uncertainty Analysis; Incomplete Reporting of Model Results

 While the authors perform a probabilistic sensitivity analysis (PSA) using Monte Carlo simulation, they do not present any base case (deterministic) results. This omission is highly problematic, as deterministic results form the foundational reference point for interpreting model behaviour and incremental comparisons. The probabilistic results that are reported are also incomplete and poorly presented — no full breakdown of QALYs, life years, or costs by adherence level is given, nor are ICERs or incremental comparisons shown.

 Furthermore, structural uncertainty is not addressed. Key assumptions — such as the static nature of adherence, the exclusive linkage between adherence and exacerbation risk, and the structure of transitions — are accepted without testing alternatives. The influence of these assumptions should be explored through scenario analyses or alternative model specifications. While the PSA addresses some degree of parameter uncertainty, the underlying model inputs are insufficiently transparent to assess the robustness or validity of the simulation.

Is the work clearly and accurately presented and does it cite the current literature?

No

If applicable, is the statistical analysis and its interpretation appropriate?

Partly

Are all the source data underlying the results available to ensure full reproducibility?

No

Is the study design appropriate and is the work technically sound?

No

Are the conclusions drawn adequately supported by the results?

No

Are sufficient details of methods and analysis provided to allow replication by others?

No

Reviewer Expertise:

Research Fellow in Pharmacoeconomics, expert in health economics modelling and research prioritisation

I confirm that I have read this submission and believe that I have an appropriate level of expertise to state that I do not consider it to be of an acceptable scientific standard, for reasons outlined above.

# Reviewer report: Reviewer response for version 2

**DOI:** 10.3310/nihropenres.15379.r38298

**Published:** 2025-11-18

**Authors:** Carlo Lazzaro

**Affiliations:** 1 University of Pavia, Pavia, Italy

**Comments on the paper:**

1. Page 2, Abstract, Conclusions: Do “economic benefits” mean savings? If that were the case, I would go with the latter.
2. Page 4, Methods:
3. Page 4, Methods: a reference is needed after “Straight Talking Asthma Information Project”.
4. Page 5, Model assumptions: “1) The length of acute exacerbations and associated hospitalizations does not affect transition probabilities outside of control status and medication adherence”. This statement seems to recall the n-1 cycle lack of memory Markov models (Sonnenberg FA, Beck JR. Markov models in medical decision making: a practical guide. Med Decis Making. 1993 Oct-Dec;13(4): 326). If that were the case, a brief explanation can help readability.
5. Page 6, Transition probabilities and treatment effectiveness: the approach followed to translate odds ratio into probabilities should be clarified (here or in the Supplemental Material). A suggestion may come from a reference quoted by Authors (Briggs A, Sculpher M, Claxton K. Decision modelling for health economic evaluation. Oxford: Oxford University Press; 2006:51).
6. Page 6, Transition probabilities and treatment effectiveness: is the “zero adherence” scenario realistic?
7. Page 6, Transition probabilities and treatment effectiveness: here or in the Supplemental Material more details about the formula used to calculate the treatment effect would be welcomed.
8. Page 7, Health state utility values and cost-effectiveness analysis: does the sentence “…all outputs are reported over a 20-year time horizon but with costs and QALYs are discounted at a rate of 3.5% per annum (NICE, 2022).” read “…all outputs are reported over a 20-year time horizon with costs and QALYs discounted at a rate of 3.5% per annum (NICE, 2022).”?
9. Page 7, Health state utility values and cost-effectiveness analysis: does “…parameter uncertainty…” mean “…joint parameter uncertainty…” (Briggs A, Sculpher M, Claxton K. Decision modelling for health economic evaluation. Oxford: Oxford University Press; 2006:144)? On a different fashion, why one or more deterministic sensitivity analyses, other than adherence scenarios, were not performed?
10. Supplementary Materials, Table S2 Transition probabilities: it is not clear why transition probabilities were not assigned a Dirichlet distribution (Briggs A, Sculpher M, Claxton K. Decision modelling for health economic evaluation. Oxford: Oxford University Press; 2006:87-88;117-118)
11. Supplementary Materials, Table S3 Health Utilities: it is not clear if these are utilities or disutilities.
12. Supplementary Materials, Table S3 Health Utilities: it is not clear if these are utilities or disutilities.
13. Supplementary Materials, Table S4: Cost of Treatment: it is not clear why costs were not included in PSA.

Is the work clearly and accurately presented and does it cite the current literature?

Partly

If applicable, is the statistical analysis and its interpretation appropriate?

Partly

Are all the source data underlying the results available to ensure full reproducibility?

Yes

Is the study design appropriate and is the work technically sound?

Yes

Are the conclusions drawn adequately supported by the results?

Yes

Are sufficient details of methods and analysis provided to allow replication by others?

Partly

Reviewer Expertise:

Pharmacoeconomics; health economics; (bio)statistics; health econometrics.

I confirm that I have read this submission and believe that I have an appropriate level of expertise to confirm that it is of an acceptable scientific standard, however I have significant reservations, as outlined above.

# Reviewer report: Reviewer response for version 2

**DOI:** 10.3310/nihropenres.15379.r38097

**Published:** 2025-11-13

**Authors:** Andrea Manfrin

**Affiliations:** 1 University of Central Lancashire, Preston, UK

Dear Editor, and Dear Authors, thank you very much for inviting me to review this manuscript titled:

Modelling the case for cost-effectiveness of interventions to improve medication adherence in patients with difficult to control asthma

 This is a very important article informing stakeholders about medication adherence in patients with difficult-to-control asthma.

 I have attached my full review below, hoping that it will help to strengthen the manuscript.

**Manuscript’s strengths**

 It focuses on an important and actual topic.

 It provides an interesting outlook on asthma

 It suggests an interesting angle to understand the conditions

**Suggestions for improving the manuscript.**

 The authors are presenting the benefits of adherence and consistent compliance. It is suggested to expand on the two different concepts to enhance the accuracy of the information.

 A paper indexed focused on adherence, defined it, as the Holy Grail. Please consider this paper for inclusion in the literature, as it may enhance the introduction and discussion.

 The study has the following aims: to assess the long-term health impact and resultant cost to the health and social care system of poor adherence to treatment, and by extension, a means of estimating the potential cost-effectiveness of interventions designed to enhance adherence

 The authors should provide the definition of the control state of the patient population based on the 2023 GINA Asthma guideline, which suggests two types of questionnaires: the ACT and ACQ.

 In this manuscript, it is unclear which questionnaire was selected, along with its respective outcomes. This is very important because the two questionnaires worked in the opposite direction.

 ACT: asthma control increases when the scores increase.

 ACQ: asthma control increases when the scores decrease. 

 As in the manuscript, the authors refer to 5 items; it seems that the manuscript considered the ACT score. The ACQ is a 7-item questionnaire. This should be clearly explained to enhance clarity for the reader's benefit.

 If the concept of control is used according to the ACT score, then the authors should clearly define the score boundaries of ACT control: not controlled, partially controlled, and controlled.

 This will add strengths to the following section: Cohort patients

 Asthma control and asthma exacerbation are two different issues. The authors should provide a clear definition for each and explain the differences between them. This will add clarity and enhance the manuscript's strength.

 Model assumption: I am unsure that adherence can be considered static over time. Several publications suggest that adherence fluctuates. Please consider this aspect during the revision and implementation of the manuscript.

 “The main problem of defining adherence, as  in this systematic review, adherence was defined using medication possession ratio (MPR) and prescription refill data, which are widely used measures of long-term medication-taking behaviour”; MRP does not imply that the patients have taken the medications.

 It might be useful for the authors to examine other papers that provide easy-to-interpret information on representing the relationship between asthma control and adherence to asthma medications.

 The following reference (60(5): 669–685.e2) is over 20 years old. It might be useful to identify more recent studies to provide more updated information on the relationship.

 The authors cited several key concepts: asthma control, asthma exacerbation, adherence, adherence in patients with difficult-to-control asthma, and effectiveness. Nonetheless, in the paper, there are no very clear definitions for each one of the above. It is suggested to revise and provide clear, updated definitions, along with their pros and cons. This should strengthen the manuscript and add clarity to readers.

**Discussion**

 Once the authors have reviewed the definitions and included them in their manuscript, they will be able to link them to the results and discussion, creating a more articulated narrative.

 Additionally, it would be worth improving the structure of the discussion section by dividing it into the following subsections: 1) summary of results, 2) comparison with the current literature, 3)  strengths and limitations, 4) impact on policies, practice and research. This will add a more consistent structure to the presentation of this very important part of the manuscript.

Is the work clearly and accurately presented and does it cite the current literature?

Partly

If applicable, is the statistical analysis and its interpretation appropriate?

Yes

Are all the source data underlying the results available to ensure full reproducibility?

Yes

Is the study design appropriate and is the work technically sound?

Yes

Are the conclusions drawn adequately supported by the results?

Partly

Are sufficient details of methods and analysis provided to allow replication by others?

Partly

Reviewer Expertise:

Health services research, effectiveness and cost-effectiveness interventions in asthma patients,  and advanced statistical analysis

I confirm that I have read this submission and believe that I have an appropriate level of expertise to confirm that it is of an acceptable scientific standard, however I have significant reservations, as outlined above.

# Reviewer report: Reviewer response for version 2

**DOI:** 10.3310/nihropenres.15379.r37876

**Published:** 2025-11-05

**Authors:** Tallys Feldens

**Affiliations:** 1 Sant'Anna School of Advanced Studies, Pisa, Italy

The paper has acknowledged its limitations and I hope in the future new research may go further in modelling the complexities that were left behind.

Is the work clearly and accurately presented and does it cite the current literature?

Yes

If applicable, is the statistical analysis and its interpretation appropriate?

Yes

Are all the source data underlying the results available to ensure full reproducibility?

Partly

Is the study design appropriate and is the work technically sound?

Yes

Are the conclusions drawn adequately supported by the results?

Partly

Are sufficient details of methods and analysis provided to allow replication by others?

Partly

Reviewer Expertise:

Health Economics

I confirm that I have read this submission and believe that I have an appropriate level of expertise to confirm that it is of an acceptable scientific standard.

# Reviewer report: Reviewer response for version 1

**DOI:** 10.3310/nihropenres.14963.r36365

**Published:** 2025-08-19

**Authors:** Tallys Feldens

**Affiliations:** 1 Sant'Anna School of Advanced Studies, Pisa, Italy

Dear authors,

 Congratulations for this very timely and important paper. I hope I can express my suggestions so the authors may improve even more the arguments and the discussion of this paper.

 Strong points

 Subject very interesting and relevant

 Clear and easy to read

 Mentioned the sources of the inputs

 Weaknesses

 I know it would introduce complexity to the model, but if you are not willing to deal with that, at least it should be mention as a limitation the role of the comorbidities in the asthma control. The adherence to the treatment is not random and probably is correlated with several factors you've briefly mentioned. One point that was not covered was the role of the comorbidities and how they could interact with the behavior of the patient during the control.

 It should be given more details about how the treatment you are considering the patients to adhere or not compares with your literature. I liked that you are using the recent guidelines from the major authority in Asthma nowadays, however, some of your key literature (Chongmelaxme, 2003) has more than 20 years and probably do not refer to the same "adherence" as you do. Please discuss thoroughly.

 Still about the same reference, it should be mention how did you adapt the systematic review results to a week cycle. 

 The choice of the weekly cycle was intelligent and I think it makes much sense for asthma control. However, I do not think you have explored the short term impacts that this cycle length allows you to. In the end, you could have used any other cycle length and the results would be the same. Please enhance this discussion in order to value your model choices.

 Asthma prevalences are different according to the age and gender, but you have used only the mean values. Would this cost-effectiveness be more impactful for any of these subpopulations?

 I am not completely convinced about the assumption of constant adherence levels. If we are hypothesizing an intervention to educate the patients, are we considering that such effects would be permanent? I think this is an assumption hard to make. I suggest you to reframe your argumentation about this, maybe including relevant literature or providing some examples.

 Please make an observation about the possible interaction of asthma cases and pollution/climate change. As we are thinking on 20y horizon, this effect is probably non-negligible. Also, when you mention the costs with the carbon footprint of the treatment, go ahead and indicate a back-of-the-envelope estimation to enhance your argumentation.

 Your discussion should have more discussion with other studies that estimated this type of CEA. You have mentioned the recent SR of Khaw, please get some of this literature and prepare a deeper discussion on the methodological similarities, assumptions and results of those papers.

Is the work clearly and accurately presented and does it cite the current literature?

Yes

If applicable, is the statistical analysis and its interpretation appropriate?

Yes

Are all the source data underlying the results available to ensure full reproducibility?

Partly

Is the study design appropriate and is the work technically sound?

Yes

Are the conclusions drawn adequately supported by the results?

Partly

Are sufficient details of methods and analysis provided to allow replication by others?

Partly

Reviewer Expertise:

Health Economics

I confirm that I have read this submission and believe that I have an appropriate level of expertise to confirm that it is of an acceptable scientific standard, however I have significant reservations, as outlined above.
